# Effect of Musk Tongxin Dropping Pill on Myocardial Remodeling and Microcirculation Dysfunction in Diabetic Cardiomyopathy

**DOI:** 10.1155/2021/6620564

**Published:** 2021-03-18

**Authors:** Jingjing Zhao, Yan Zhou, Huahua Liu, Zhaohai Zheng, Shuqing Liu, Jiahao Peng, Hangyuan Guo, Weiliang Tang, Fang Peng

**Affiliations:** ^1^Department of Cardiology, Shaoxing People's Hospital (Shaoxing Hospital, Zhejiang University School of Medicine), Shaoxing, Zhejiang 312000, China; ^2^Zhejiang University School of Medicine, Hangzhou, Zhejiang 310000, China; ^3^Cardiovascular Research Center, School of Basic Medical Sciences, Xi'an Jiaotong University Health Science Center, Xi'an 710000, China; ^4^Shaoxing University School of Medicine, Shaoxing, Zhejiang 312000, China; ^5^Loma Linda University School of Public Health, 24951 Circle Dr, Loma Linda, CA 92354, USA

## Abstract

**Objective:**

To explore the effect of Musk Tongxin Dropping Pill (MTDP) on myocardial remodeling and microcirculation dysfunction in diabetic cardiomyopathy (DCM).

**Methods:**

Forty male SD rats were randomly divided into control group (control group, *n* = 10), DCM model group (DCM group, *n* = 10), DCM model + pioglitazone group (DCM + PLZ group, *n* = 10), and DCM model + MTDP group (DCM + MTDP group, *n* = 10). An intraperitoneal single injection of 65 mg/kg streptozotocin (STZ) was used to establish rat model of DCM and the rats in control group were treated with the same dose of sodium citrate buffer solution. DCM + PLZ group was treated with 3 mg/kg/d PLZ by ig after modeling, DCM + MTDP group was treated with 22 mg/kg/d MTDP by ig, and DCM group was treated with 2 ml/kg/d sodium carboxymethyl cellulose (CMC-Na) by ig. The general condition of rats was continuously observed. After intervening for 3 weeks, the random blood glucose of rats was detected by tail vein, and the echocardiography examination was performed. Blood specimens were collected from the abdominal aorta, serum nitric oxide (NO) and endothelin-1 (ET-1) were detected to estimate endothelial function, and tumor necrosis factor *α* (TNF-*α*), interleukin 6 (IL-6), IL-1*β*, malondialdehyde (MDA), and superoxide dismutase (SOD) were detected to observe the changes of inflammation and oxidative stress indexes. The heart mass index (HMI) was calculated through the ratio of heart mass (HM) to the corresponding body mass (BM). Myocardial pathological tissue staining was performed.

**Results:**

Compared with control group, blood glucose in other three groups was higher. Left ventricular end systolic diameter (LVSD) and left ventricular end diastolic diameter (LVDD) in DCM group showed a significant increase, while left ventricular ejection fraction (LVEF) and heart rate (HR) in this group displayed an obvious decrease (*P* < 0.01). BM and HM in DCM group exhibited a reduction, and HM/BM × 10^3^ revealed an apparent increase (*P* < 0.01). The levels of serum NO and SOD were distinctly downregulated (*P* < 0.01), and the levels of ET-1, MDA, TNF-*α*, IL-1*β*, and IL-6 were remarkably upregulated (*P* < 0.01). Compared with DCM group, a significant decrease was observed in LVSD and LVDD in DCM + MTDP group, while LVEF and HR obviously increased (*P* < 0.05). BM and HM indicated an apparent increase, but HM/BM ×10^3^ reduced distinctly (*P* < 0.01). The levels of serum NO and SOD were markedly upregulated (*P* < 0.05), and the levels of ET-1, MDA, TNF-*α*, IL-1*β*, and IL-6 were significantly downregulated (*P* < 0.05). HE staining showed that myocardial cells arranged neatly in the control group but not in the DCM group. The intercellular space between myocardial cells in DCM group increased, accompanied by damage of myocardial fibers and infiltration of inflammatory cells. Masson staining displayed an increase in interstitial collagen fibers in DCM group. Carstairs staining showed that microembolization occurred in the myocardium in DCM group, while in DCM + MTDP and DCM + PLZ groups the corresponding myocardial pathological changes were significantly improved.

**Conclusions:**

MTDP might show a positive effect on myocardial remodeling and microcirculation dysfunction in DCM rats.

## 1. Introduction

With the development of society, the prevalence of diabetes mellitus (DM) has increased dramatically. According to the prediction of the International Diabetes League, by 2035, the number of pre-DM patients in the world will reach 471 million, and the number of DM patients will reach 592 million [1]. DM not only seriously affects the regulation of blood glucose level, but also significantly increases the occurrence of other chronic complications, involving the heart, brain, kidney, circulation, nerve, and other systems. These influences can lead to coronary heart disease, cerebral infarction, peripheral atherosclerosis, and other diseases, and seriously impact the health and quality of life of patients. Diabetic cardiomyopathy (DCM) is one of the main and independent chronic complications of DM [2]. DCM is a cardiac insufficiency induced by DM, and the incidence rate is as high as 16.9% [3] in DM patients. DCM is closely related to the high incidence and mortality of heart failure in DM patients [4–6]. de Simone et al. [7] found that the risk of heart failure in diabetic patients without hypertension and myocardial infarction is increasing gradually. However, the pathogenesis of DCM has not been fully understood, and more interventions of DCM need to be further explored.

At present, western medicine has made more positive achievements in the treatment of DCM, such as endogenous insulin treatment, anti-oxidative stress treatment, inhibition of RAAS system, anti-heart failure treatment, etc. In clinical treatment, doctors mainly use drugs such as blood glucose regulators [8, 9], ascorbic acid, ACEI/ARB, *β* receptor blocker, etc. However, each of these drugs usually only plays a single role through a few of simple mechanisms as mentioned above. And for DCM, a disease with complex mechanism, it is more meaningful to explore a way of comprehensive intervention from many aspects. At this time, traditional Chinese medicine prescriptions have shown certain advantages in comprehensive treatment, especially Musk Tongxin Dropping Pill (MTDP), which have multiple functions such as improving blood circulation, removing blood stasis, and reinforcing functional activities of heart.

In recent years, we preliminarily confirmed that MTDP could improve coronary slow blood flow by anti-inflammatory, anti-oxidation, protecting vascular endothelium, and improving microcirculation function. However, it is not clear whether MTDP has similar effect on DCM. For further exploration, we discussed this matter in this study.

## 2. Materials and Methods

### 2.1. Materials

Animals: 40 specific-pathogen-free (SPF) male SD rats weighing about 150–200 g were all purchased from Zhejiang Academy of Medical Sciences (license number: SCXK (Zhejiang) 2019–0002), all of them passed the inspection of Zhejiang Experimental Animal Quality Supervision and Monitoring Station (license number:1905080021), and they were raised in the laboratory animal feeding room of Shaoxing Hospital of Zhejiang University (license number: SYXK (Zhejiang) 2017–0007).

Reagents were as follows: MTDP (powder provided by Inner Mongolia Kangenbei Pharmaceutical Co., Ltd.), streptozotocin (STZ), sodium citrate buffer, pioglitazone (PLZ), sodium carboxymethyl cellulose (CMC-Na), nitric oxide (NO) test kit, all related enzyme-linked immunosorbent assay (ELISA) test kits, hematoxylin-eosin (HE) staining kit, Masson staining kit, Carstairs staining kit, 1% pentobarbital sodium, and sevoflurane.

### 2.2. Modeling and Intervention

The protocol was approved by the Ethics Committee of Shaoxing People's Hospital (the Ethics Committee of Shaoxing People's Hospital consists of 16 members, including one chairman and two vice-chairmen).

A total of 40 male SD rats were randomly divided into 4 groups: control group, DCM model group (DCM group), DCM model + MTDP group (DCM + MTDP group), and DCM model + PLZ group (DCM + PLZ group); there were 10 rats in each group. An intraperitoneal single injection of 65 mg/kg STZ (sodium citrate buffer solution as solvent) was used to establish rat model of DCM; the rats in control group were treated with the same dose of sodium citrate buffer solution.

One week after a single intraperitoneal injection of STZ, the vein blood was taken from the tail vein of the rats, and the random blood glucose of the rats was detected. No disease or death occurred in the rats, and the blood glucose of the 30 SD rats was more than 30 mmol/L (if the blood glucose was more than 16.7 mmol/l, the model was successful), indicating that all the models were successful.

Then, 22 mg/kg MTDP (0.5% CMC-Na as solvent) and 5 mg/kg PLZ (0.5% CMC-Na as solvent) were given to DCM + MTDP group and DCM + PLZ group, respectively, 2 ml/kg CMC-Na was given to DCM group; all of them were given by intragastric gavage once a day for 3 weeks. During the experiment, the amount of drinking water, the wetting condition of bedding, and the activity of rats in each group were observed every day. No disease or death occurred in the rats. The oral dose of rats was calculated according to the adult oral dose/body weight *∗* 6.3 mg/kg, and the random blood glucose of rats was detected by tail vein again at the end of the gavage intervention.

After fasting and water prohibition for 12 hours, the rats were anesthetized by intraperitoneal injection of 1% pentobarbital sodium (2 ml/kg) (sevoflurane inhalation anesthesia could be performed if necessary). The body mass (BM) was recorded and echocardiography examination was performed. Then, the blood samples from abdominal aorta were collected and the hearts were isolated under the help of surgical operation. And the whole heart mass (HM) was recorded; the hearts were fixed with 4% formaldehyde solution at 4°C for pathological examination.

### 2.3. Echocardiography Examination

The echocardiography examination was carried out by the same operator. The data needed in this study mainly include left ventricular end systolic diameter (LVSD), left ventricular end diastolic diameter (LVDD), left ventricular ejection fraction (LVEF), and heart rate (HR).

### 2.4. Detection of Endothelial Function

According to the specific operation steps of NO detection kit, NO content in serum was detected (nitrate reductase method). The level of endothelin-1 (ET-1) in serum was detected according to the specific operation of ELISA kit.

### 2.5. Detection of Inflammation and Oxidative Stress

The levels of tumor necrosis factor *α* (TNF-*α*), Interleukin 6 (IL-6), IL-1*β*, and malondialdehyde (MDA) in serum were detected by ELISA, and the activity of superoxide dismutase (SOD) in serum was determined by visible spectrophotometry.

### 2.6. Calculation of Heart Mass Index

The ratio of HM to the corresponding BM was calculated, that is, the heart mass index (HMI).

### 2.7. Myocardial Pathological Tissue Staining

HE staining kit, Masson staining kit, and Carstairs staining were carried out.

### 2.8. Statistical Methods

Spss 24.0 and Prism 7.0 software were used for statistical analysis. The data were analyzed by the means of mean ± standard deviation, and the comparison between groups was tested by Tukey's multiple comparisons test. *P* < 0.05 was statistically significant.

## 3. Results

### 3.1. Echocardiographic Results

Compared with control group, LVSD and LVDD of SD rats in DCM group showed a significant increase (*P* < 0.01), where the mean value of LVSD and LVDD was 3.8 mm and 6.7 mm, respectively. A significant decrease was observed in LVSD and LVDD in DCM + MTDP group compared with DCM group (*P* < 0.01); mean values were 3.0 mm and 6.0 mm, respectively.

Compared with control group, LVEF and HR in DCM group displayed an obvious decrease (*P* < 0.01), while LVEF in DCM + MTDP group significantly increased (*P* < 0.05) and HR obviously increased (*P* < 0.01) (Table 1).

### 3.2. Blood Glucose, Body Mass, and Heart Mass Index

Compared with control group, the BM and HM in DCM group exhibited a reduction (*P* < 0.01), while the blood glucose of the other three groups was higher. Compared with DCM group, the BM and HM in DCM + MTDP group indicated an apparent increase (*P* < 0.01).

Different from the control group, the HM/BM ×10^3^ in DCM group revealed an apparent increase (*P* < 0.01), and compared with the DCM group, the HM/BM ×10^3^ in DCM + MTDP group reduced distinctly (*P* < 0.01) (Table 2).

### 3.3. Vascular Endothelial Function

**Figure 1:**
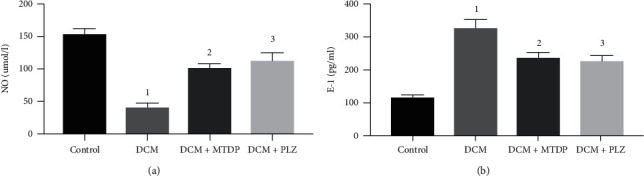
NO and ET-1 in each group. NO: nitric oxide; ET-1: endothelin-1; DCM: diabetic cardiomyopathy; MTDP: Musk Tongxin Dropping Pills; PLZ: pioglitazone. Compared with control group, ^1^*P* < 0.01; compared with DCM group, ^2^*P* < 0.01 and ^3^*P* < 0.01.

The results of serum endothelial function test showed that, compared with control group, the level of NO in DCM group was distinctly downregulated (*P* < 0.01), and the level of ET-1 was remarkably upregulated (*P* < 0.01). Compared with DCM group, the level of NO in DCM + MTDP group and DCM + PLZ group was markedly upregulated, and the level of ET-1 was significantly downregulated (*P* < 0.01) (Figure 1).

### 3.4. Inflammatory Factors

**Figure 2:**
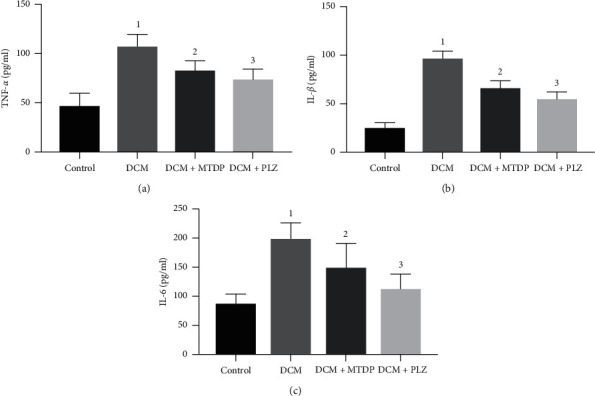
TNF-*α*, IL-1*β*, IL-6 in each group. TNF: tumor necrosis factor; IL: interleukin; DCM: diabetic cardiomyopathy; MTDP: Musk Tongxin Dropping Pills; PLZ: pioglitazone. Compared with control group, ^1^*P* < 0.01; compared with DCM group, ^2^*P* < 0.01 and ^3^*P* < 0.01.

The results of serum inflammatory factors in each group showed that, compared with control group, the levels of TNF-*α*, IL-1*β*, and IL-6 in DCM group were significantly higher (*P* < 0.01), while the levels of TNF-*α*, IL-1*β*, and IL-6 in DCM + MTDP group and DCM + PLZ group were significantly lower (*P* < 0.01) compared with DCM group (Figure 2).

### 3.5. Indexes of Oxidative Stress

**Figure 3:**
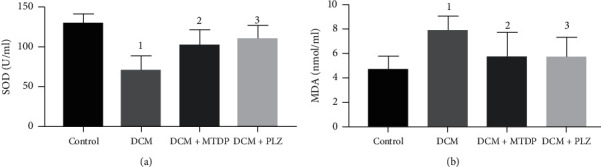
SOD, MDA in each group. SOD: superoxide dismutase; MDA: malondialdehyde; DCM: diabetic cardiomyopathy; MTDP: Musk Tongxin Dropping Pills; PLZ: pioglitazone. Compared with control group, ^1^*P* < 0.01; compared with DCM group, ^2^*P* < 0.05 and ^3^*P* < 0.05.

The results of serum oxidative stress indexes showed that, compared with control group, the level of serum SOD in DCM group was significantly lower (*P* < 0.01), MDA was significantly higher (*P* < 0.01), compared with DCM group, the level of SOD in DCM + MTDP group and DCM + PLZ group was significantly higher (*P* < 0.05), and MDA was significantly lower (*P* < 0.05) (Figure 3).

### 3.6. Myocardial Pathological Staining

#### 3.6.1. HE Staining

**Figure 4:**
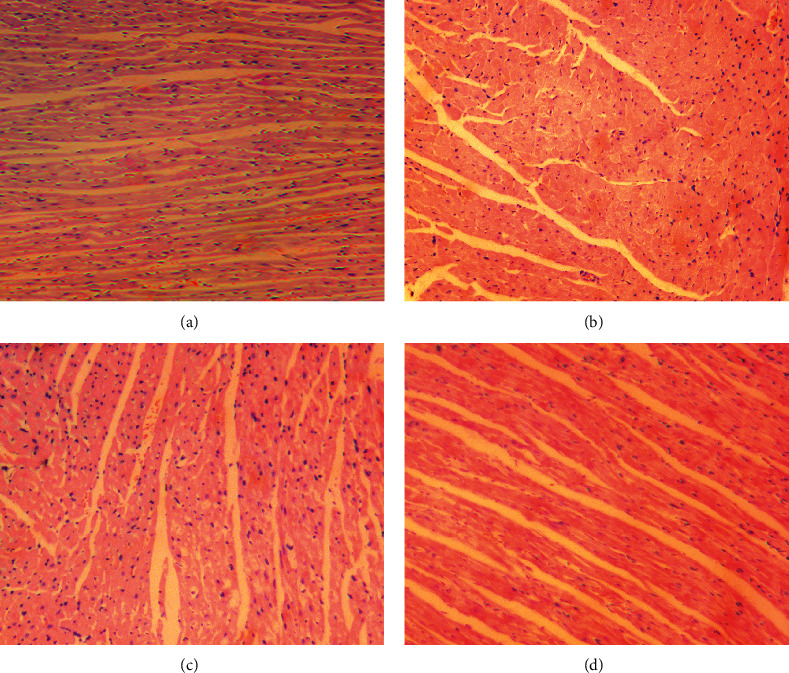
The result of HE staining of cardiomyocytes (×200 magnification, bar = 100 um). (a) Control group, (b) DCM group, (c) DCM + MTDP group, and (d) DCM + PLZ group.

The results of HE staining indicated that the cardiomyocytes in control group arranged neatly but not in DCM group, and the intercellular space between myocardial cells in DCM group increased, accompanied by damage of myocardial fibers and infiltration of inflammatory cells, while in DCM + MTDP and DCM + PLZ groups, the myocardial pathological changes were significantly improved (Figure 4).

#### 3.6.2. Masson Staining

**Figure 5:**
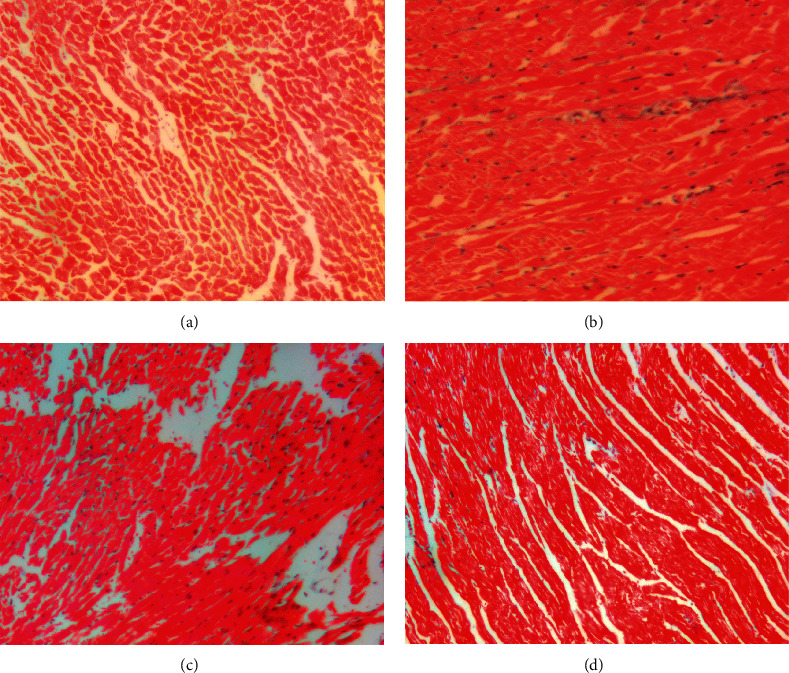
The result of Masson staining of cardiomyocytes (×200 magnification, bar = 100 um); blue area indicates collagen fiber. (a) Control group, (b) DCM group, (c) DCM + MTDP group, and (d) DCM + PLZ group.

Masson staining displayed an increase in interstitial collagen fiber (blue area) in DCM group, while in DCM + MTDP and DCM + PLZ groups, the myocardial pathological changes were significantly improved (Figure 5).

#### 3.6.3. Carstairs Staining

**Figure 6:**
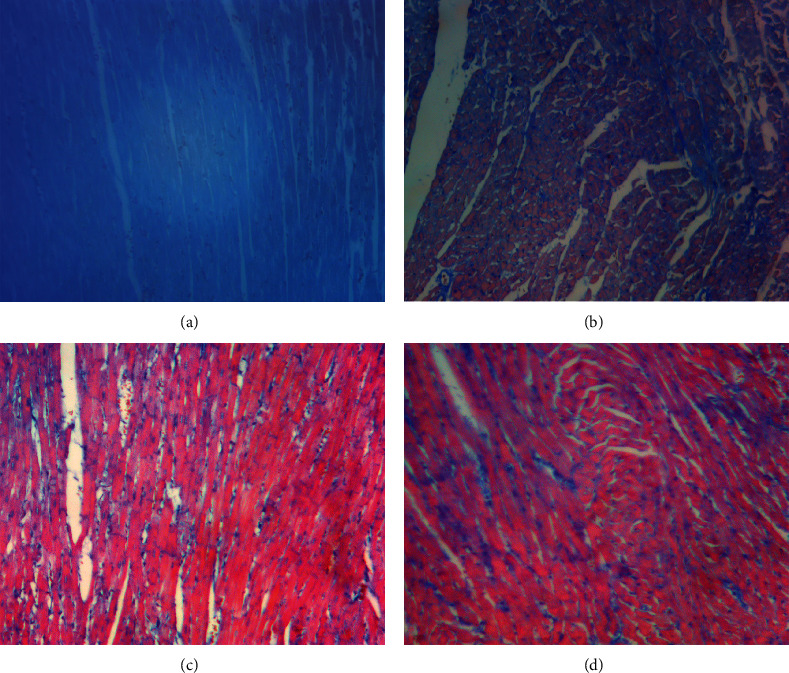
The result of Carstairs staining of cardiomyocytes (×200 magnification, bar = 100 um); blue aggregation area represents microembolism. (a) Control group, (b) DCM group, (c) DCM + MTDP group, and (d) DCM + PLZ group.

The results of Carstairs staining showed that myocardial microembolism occurred in DCM group, while in DCM + MTDP and DCM + PLZ groups, the myocardial pathological changes were significantly improved (Figure 6).

## 4. Discussion

The Framingham Heart Study and The Strong Heart Study both have reported that DM is an independent risk factor for heart failure [10, 11]. At the moment, it has been found that DM can directly damage the heart muscle, cause cardiac hypertrophy, bring about systolic and diastolic dysfunction, and finally lead to heart failure. Cardiovascular complications are the main cause of increased incidence rate and mortality of DM patients [12]. DCM is now considered as an independent complication of DM.

DCM is characterized by systolic and diastolic dysfunction. Its main pathological changes include myocardial remodeling, coronary microcirculation dysfunction, and so on [13]. The mechanisms involved in the pathogenesis include oxidative stress, inflammatory response, myocardial cell apoptosis, myocardial fibrosis, vascular endothelial dysfunction, coronary microvascular embolism, etc. Studies [14] have shown that, in DM patients, due to glucose utilization disorders, cardiac myocytes mostly obtain energy through lipid metabolism. In this process, lipid peroxidation occurs and a large number of reactive oxide species (ROS) are released, which result in oxidative stress. At the same time, on account of the stimulation of high glucose, inflammatory factors such as nuclear factor kappa B (NF-*κ*B), TNF-*α*, and IL-6 are significantly increased in the cardiac tissue of patients, suggesting that inflammatory response is involved in the occurrence and development of DCM [15, 16]. Under the action of high glucose, oxidative stress, and inflammatory factors, many apoptotic signal pathways are activated, which induce cardiomyocyte apoptosis. Pathological changes such as the imbalance of matrix metalloproteinase/tissue inhibitor of metalloproteinase (MMP/TIMP) and the abnormal deposition of extracellular matrix can lead to myocardial fibrosis and myocardial remodeling [15]. In addition, the disorder of glucose and lipid metabolism, oxidative stress, and inflammatory reaction can cause the damage and dysfunction of endothelial cell. This process can result in diastolic dysfunction of the coronary artery system, abnormal aggregation of platelets, microvascular thrombosis, and myocardial ischemia [17]. In conclusion, the occurrence and development of DCM are complex processes under the interaction of various mechanisms. And it might be conducive to the improvement of DCM to explore a way which plays a positive role via the multiple mechanisms mentioned above.

MTDP, which is a Chinese traditional medicine, is commonly used in clinical treatment of cardiovascular diseases. The components of MTDP mainly include musk, bezoar, ginsenoside, salvia miltiorrhiza, venenum bufonis, bear bile powder, and borneol. The researches have shown several multiple functions of MTDP. Xiong et al. [18] demonstrated that, in ApoE (-/-) mouse model, MTDP attenuated atherosclerotic lesions, and MTDP displayed multi-targeting roles in biochemical, molecular, and pathological aspects of atherosclerosis implicating inflammation, oxidative stress, lipid regulation, and fibrosis. Zhang et al. [19] revealed that MTDP markedly improved peripheral microvascular blood flow and microcirculation; the increased expression of cystathionine-*γ*-lyase might be a possible mechanism. Qi et al. [20] found that MTDP protected against isoproterenol-induced myocardial ischemic injury via an ERK1/2 signaling pathway. In order to explore the potential effect of MTDP on DCM, we made DCM rat model via one-time intraperitoneal injection of STZ and treated them with MTDP.

In DCM group, an impairment of systolic and diastolic function of left ventricle was observed, manifested as an increase of LVSD and LVDD and a decrease of LVEF, which were consistent with the cardiac pathologic changes of DCM reported previously [21, 22]. Compared with DCM group, the LVEF of rats treated with MTDP obviously increased accompanied by a reduction of LVSD, LVDD, and HMI. It showed that MTDP might play a positive role in the structure and function of heart in DCM.

We found that, in MTDP intervention group, the levels of serum NO and SOD were markedly upregulated, and the levels of ET-1, MDA, TNF-*α*, IL-1*β*, and IL-6 were significantly downregulated. The damage of vascular function in DCM is related to endothelial dysfunction, changes of humoral factors, and fibrosis of vascular wall. Under the condition of hyperglycemia, the formation of advanced glycation end products (AGEs) increases; AGEs binding to AGE receptors (RAGE) can cause various damages in endothelial cells, such as increasing endothelial cell permeability, inhibiting the activity of endothelial nitric oxide synthetase, affecting coagulation system; in addition, they can increase the production of ROS and activate nicotinamide adenine dinucleotide phosphate (NADPH) oxidase and NF-*κ*B [17, 23]. AGEs can lead to crosslinks in collagen molecules, accompanied by weakening the ability of collagen to be degraded, causing increased fibrosis with following increased myocardial stiffness and damaged cardiac relaxation [24]. The expression of RAGE is induced by oxidative stress in the hearts of DM [25]. Meanwhile, the disordered expression of vascular active factors and vascular endothelial growth factors can cause the dysfunction of endothelial and vasoconstriction, and the upregulation of various vasoconstrictors such as ET-1 and prostaglandin can aggravate the phenomenon. At the same time, insufficient bioavailability of vasodilator of NO can damage endothelium-dependent vasodilation [23]. The level of fibrosis around blood vessels and blood vessel walls is more serious than that in myocardial tissue; vascular damage caused by collagen fiber III is the most significant accumulation of collagen in vascular fibrosis.

In the development of DCM, inflammation and oxidative stress play an irreplaceable role [26]. Inflammatory manifestations in the myocardium of DCM include increased expression of Intercellular cell adhesion molecule-1 (ICAM-1) and vascular cell adhesion molecule-1 (VCAM-1), increased infiltration of leukocytes and macrophages, and increased expression of inflammatory cytokines (TNF-*α*, TGF-*β*1, IL-6, and IL-18). Additionally, SOD is a vital antioxidant enzyme, and its activity can indirectly reveal the ability of scavenging oxygen free radicals [27]. And MDA, one of the decomposition products of peroxidized polyunsaturated fatty acids, was deemed to be sensitive for the estimation of oxidative stress [28]. A significant increase of mitochondrial superoxide was found in DM mice model. However, inflammatory response and oxidative stress are complementary. ROS can damage the DNA of cardiomyocytes, change the expression of genes, and affect the expression of pro-inflammatory factors. In the environment of high glucose, a variety of signal pathways such as inflammatory factors, oxidative stress, and other factors are activated; they can cause abnormal deposition of extracellular matrix and myocardial fibrosis and lead to myocardial remodeling [15]. The fibrosis of vascular walls and peripheral vessels in DCM is often serious, which can aggravate the severity of vascular damage and result in vasomotor abnormalities; it can also change the permeability of vascular walls. Adameova and Dhalla [17] had found that glucose metabolism disorder, oxidative stress, and inflammatory response could cause the injury and dysfunction of endothelial cells and the exposure of collagen fibers under the endothelium. These processes could cause abnormal aggregation of platelets and microvascular thrombosis of coronaries, resulting in microcirculation disorders. The decrease of blood supply after microcirculation dysfunction can lead to the injury of middle and small arteries, decrease their compliance, and further aggravate myocardial fibrosis and cardiac insufficiency. Therefore, the results of this study are consistent with the previous findings. MTDP might be effective in the improvement of endothelial dysfunction, anti-inflammatory, and anti-oxidation in DCM.

Similarly, as is put in our study, HE, Masson, and Carstairs pathological staining showed that the intervention of MTDP could improve the arrangement of disordered cardiac myocytes, the fibrosis and microembolization of myocardium.

### 4.1. Study Limitations

The current study presented several limitations. Firstly, the conclusion of this study was only based on animal research, not applied in clinical research to observe the efficacy, Secondly, the specific monomers and pathways involved in the effect of MTDP had not been thoroughly studied. Thirdly, the sample size was relatively small in this study. Finally, the technique of pathological staining needed to be improved. As a result, based on the existing research results, we will surmount the extant problems, continue to explore the role of MTDP in the prevention and treatment of DCM, and explore ways to improve the prognosis of DCM.

## 5. Conclusion

MTDP might show a positive effect on myocardial remodeling and microcirculation dysfunction in DCM rats. The potential mechanism of MTDP might be related to improving endothelial function and oxidative stress, alleviating inflammatory reaction, ameliorating myocardial fibrosis and microembolization, and improving LVEF and HMI.

## Figures and Tables

**Table 1:** Cardiac function of rats in each group (*x* ± *s*, *n* = 10).

Group	LVSD (mm)	LVDD (mm)	LVEF (%)	HR (bpm)
Control	2.4 ± 0.3	5.8 ± 0.4	88 ± 3	381 ± 13
DCM	3.8 ± 0.7^a^	6.7 ± 0.4^a^	74 ± 9^a^	249 ± 16^a^
DCM + MTDP	3.0 ± 0.2^b^	6.0 ± 0.5^b^	81 ± 3^c^	338 ± 22^b^
DCM + PLZ	2.8 ± 0.3^b^	5.8 ± 0.5^b^	81 ± 3^c^	330 ± 22^b^

DCM: diabetic cardiomyopathy; MTDP: Musk Tongxin Dropping Pills; PLZ: pioglitazone; LVSD: left ventricular end-systolic diameter; LVDD: left ventricular end-diastolic diameter; LVEF: left ventricular ejection fraction; HR: heart rate. Compared with control group, ^a^*P* < 0.01; compared with DCM group, ^b^*P* < 0.01; compared with DCM group, ^c^*P* < 0.05.

**Table 2:** Body mass, heart mass, and random blood glucose in each group (*x* ± *s*, *n* = 10).

Group	BM (g)	HM (g)	HM/BM ×10^3^	GLU (mmol/l)
Control	378 ± 10	1.21 ± 0.01	3.20 ± 0.09	6.18 ± 0.58
DCM	235 ± 10^a^	1.00 ± 0.02^a^	4.27 ± 0.13^a^	>26.2
DCM + MTDP	290 ± 38^b^	1.07 ± 0.06^b^	3.73 ± 0.42^b^	>17.4
DCM + PLZ	298 ± 45^b^	1.10 ± 0.06^b^	3.77 ± 0.51^b^	>22.1

DCM: diabetic cardiomyopathy; MTDP: Musk Tongxin Dropping Pills; PLZ: pioglitazone; BM: body mass; HM: heart mass; GLU: glucose. Compared with control group, ^a^*P* < 0.01; compared with DCM group, ^b^*P* < 0.01.

## Data Availability

The data used to support the study are available from the corresponding authors Weiliang Tang (e-mail: twl-sxyz@163.com) and Fang Peng (e-mail: sxrmyypf@126.com).

## Abbreviations

ACEI: Angiotensin-converting enzyme inhibitors
AGE: Advanced glycation end product
AGEs: Advanced glycation end products
ApoE: Apolipoprotein E
ARB: Angiotensin receptor blockers
BM: Body mass
CMC-Na: Sodium carboxymethyl cellulose
DCM: Diabetic cardiomyopathy
DM: Diabetes mellitus
DNA: Deoxyribonucleic acid
ELISA: Enzyme-linked immunosorbent assay
ERK: Extracellular regulated protein kinases
ET-1: Endothelin-1
GLU: Glucose
HE: Hematoxylin-eosin
HM: Heart mass
HMI: Heart mass index
HR: Heart rate
ICAM-1: Intercellular cell adhesion molecule-1
ig: Intragastric gavage
IL: Interleukin
IL-18: Interleukin-18
IL-1*β*: Interleukin-1*β*
IL-6: Interleukin-6
LVDD: Left ventricular end-diastolic diameter
LVEF: Left ventricular ejection fraction
LVSD: Left ventricular end-systolic diameter
MDA: Malondialdehyde
MMP: Matrix metalloproteinase
MTDP: Musk Tongxin Dropping Pill
NADPH: Nicotinamide adenine dinucleotide phosphate
NF-*κ*B: Nuclear factor kappa B
NO: Nitric oxide
PLZ: Pioglitazone
RAAS: Renin angiotensin aldosterone system
RAGE: Receptor for advanced glycation end product
ROS: Reactive oxide species
SD: Sprague Dawley
SOD: Superoxide dismutase
SPF: Specific-pathogen-free
SPSS: Statistical Product and Service Solutions
STZ: Streptozotocin
TGF-*β*1: Transforming growth factor-*β*1
TIMP: Tissue inhibitor of metalloproteinase
TNF: Tumor necrosis factor
TNF-*α*: Tumor necrosis factor-*α*
VCAM-1: Vascular cell adhesion molecule-1.
